# Small Non-coding RNAs Govern Mammary Gland Tumorigenesis

**DOI:** 10.1007/s10911-012-9246-4

**Published:** 2012-03-01

**Authors:** Zuoren Yu, Richard G. Pestell

**Affiliations:** 1 Research Center for Translational Medicine, East Hospital, Tongji University School of Medicine, Shanghai, 200120 China; 2 Department of Cancer Biology, Thomas Jefferson University, 233 South 10th St., Philadelphia, PA 19107 USA; 3 Kimmel Cancer Center, Department of Cancer Biology, Thomas Jefferson University, 233 10th Street, BLSB RM 1050, Philadelphia, PA 19107 USA

**Keywords:** Non-coding RNA, miRNA, Breast cancer, Tumorigenesis, Cell cycle

## Abstract

Small non-coding RNAs include siRNA, miRNA, piRNA and snoRNA. The involvement of miRNAs in the regulation of mammary gland tumorigenesis has been widely studied while the role for other small non-coding RNAs remains unclear. Here we summarize the involvement of miRNA in breast cancer onset and progression through regulating the cell cycle and cellular proliferation. The regulation of breast cancer stem cells and tumor regeneration by miRNA is reviewed. In addition, the emerging evidence demonstrating the involvement of piRNA and snoRNA in breast cancer is briefly described.

## Introduction

Small non-coding RNAs are transcribed into mRNA but remain untranslated in eukaryotic cells. They include siRNA (small interfering RNA), miRNA (microRNA), piRNA (piwi-interacting RNA) and snoRNA (small nucleolar RNA). miRNAs are a class of multifunctional singled-stranded small RNA which are ~20 nt in length and regulate the stability or translational efficiency of targeted messenger RNA depending on the base-pairing complementarity between the miRNA and its target mRNA [1, 2]. Although over 1,000 miRNA sequences have been identified from the tissues or cells of human origin and other species, as many as 1,000 to 10,000 miRNAs per genome have been predicted [3, 4]. miRNAs regulate a broad range of biological processes including timing of development, cell cycle progression, stem cell self-renewal, differentiation, cancer initiation, cancer cell proliferation, metastasis and apoptosis [5–11].

Cancer is caused by multiple processes including uncontrolled cellular proliferation and inappropriate survival of apoptotic cells [12]. Many regulatory factors switch on or off genes that govern cell division and direct cellular proliferation. miRNAs regulate gene expression and play important roles in the onset and progression of tumorigenesis. Breast cancer is the most common cancer among women. Emerging evidence demonstrates the involvement of miRNA in mammary gland tumorigenesis, functioning either as tumor suppressors or oncogenes [9]. Although the current treatment of radiation therapy, chemotherapy and hormone therapy slow mammary gland tumor growth, prolong survival and improve the quality of patients’ life, metastatic breast cancer still remains incurable due to our limited understanding of the molecular mechanisms through which tumorigenesis and metastasis occur. As small non-coding RNAs regulate gene expression and tumorigenesis, they may represent a novel cancer therapy.

## Aberrant Expression of miRNA in Breast Cancer

Unlike mRNA, miRNAs are transcribed but never translated. Some miRNAs are transcribed from non-coding regions between genes, deriving from independent transcription unit. Other miRNAs are transcribed together with coding mRNAs from the coding region of the genome, deriving from the introns of gene transcripts [13, 14]. miRNA gene copy number gain/loss and miRNA gene mutation have been observed in breast cancer resulting in the aberrant expression of miRNA. The first study about the altered expression of miRNAs in human breast cancer patients and human breast cancer cell lines was reported in 2005 by Lorio et al., in which 29 miRNAs were identified with aberrant expression based on microarray and northern blot analysis of 76 breast tumor samples and 14 human breast cell lines [15]. Zhang and colleagues analyzed 283 human miRNA genes on 55 human breast primary tumors and 18 human breast cancer cell lines using array-based comparative genomic hybridization. The results demonstrated a high frequency (~72.8%) of gene copy number abnormality in miRNA-containing regions in human breast cancer [16]. Wang et al. collected 68 patients with newly diagnosed breast cancer and examined the expression of selected miRNAs in tumor and adjacent non-tumor tissues. miR-21, miR-106a and miR-155 were significantly over-expressed in the tumor specimens compared with normal controls, whereas miR-126, miR-199a and miR-335 were significantly decreased in expression in the tumor samples [17]. Our studies of the miR-17-92 cluster demonstrated decreased expression of miR-17/20 in human breast cancer specimens compared with matching normal breast tissue from the same patient [18]. Subsequent analysis identified reduced miR-17/20 expression in node-positive compared with node-negative breast cancers and demonstrated that miR-17/20 inhibited breast cancer cell migration and invasion via a heterotypic signaling [19].

Although the tendency for a global decrease of miRNA expression in human cancers originally suggested a general tumor suppressor function of miRNAs [20], subsequent studies showing the aberrant expression of specific miRNAs in breast cancer suggest miRNA-specific roles in breast cancer onset and progression.

## miRNA Regulation of Cell Cycle and Mammary Gland Tumorigenesis

**Fig. 1:**
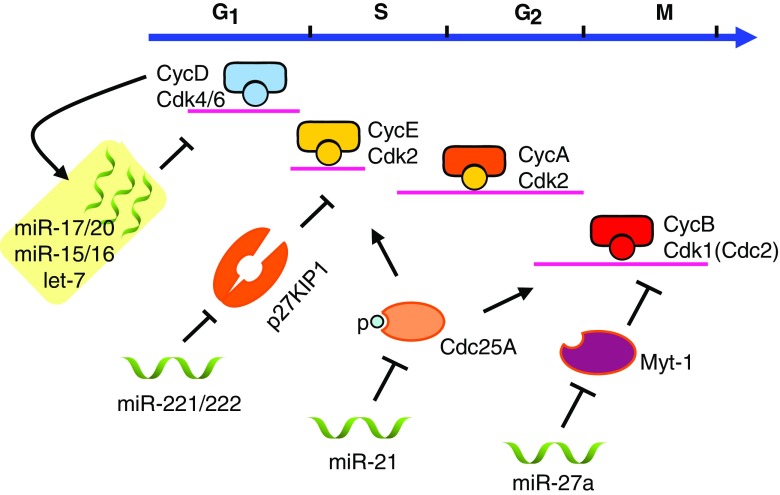
miRNA regulation of mammary gland tumorigenesis in control of the cell cycle. Through targeting different genes and different cyclin/CDK complexes, miR-17/20 and let-7 regulate the G_1_-S transition; miR-21 and miR-27a regulate the G_2_-M checkpoint

Many distinct miRNAs have been shown to regulate breast cancer cell proliferation, apoptosis, cancer stem cell expansion, and tumorigenesis. miRNA may function as either tumor suppressors or oncogenes depending on the cell type, culture conditions, target genes and pathway. The involvement of miRNA in mammary gland tumorigenesis has been reviewed recently [21, 22]. Le et al. described the expression pattern and regulatory network of key miRNAs in breast cancer, including let-7, miR-34, miR-125, miR-200 family, miR-205, miR-21, miR-10 and the miR-17-92 cluster [22]. Adams et al. reviewed the miRNA regulation of estrogen signaling pathway and ErbB2/HER signaling pathway in breast cancer [21]. The understanding of how miRNAs are involved in breast cancer through regulating the cell cycle remains rudimentary. Herein we summarize the recent literature and research progress on the mechanism by which miRNAs regulate the breast cancer cell cycle and cellular proliferation (Fig. 1).

*Cyclin D1* is either overexpressed or amplified in ~50% of breast cancer. The abundance of cyclin D1 is rate-limiting in breast cancer cellular proliferation and G_1_-S phase transition [23, 24]. In addition, *cyclin D1* is a critical downstream target of ErbB2-, Ras- and β-catenin- induced breast cancers, and is sufficient for the induction of mammary tumors when targeted to the mammary gland of mice. Antisense inhibition of *cyclin D1* expression in vivo suppressed the growth of NeuT-transformed mammary adenocarcinoma cells in nude mice [25]. Conserved sequences of the *cyclin D1* 3′UTR contain potential binding sites for multiple miRNAs including miR-17/20/106, miR-15/16, miR-23 and let-7. miR-17/20 binds the *cyclin D1* 3′UTR, inhibiting the expression of *cyclin D1*, resulting in cell cycle arrest at the G_1_ phase and suppression of MCF-7 cell proliferation [18, 26]. The regulation of *cyclin D1* expression by miR-17-92, as well as miR-15/16, was confirmed by Deshpande et al. [26]. The let-7 family functions as a tumor suppressor in a variety of cancers including lung [27], colon [28], ovarian [29] and breast cancer [30]. Schultz et al. demonstrated the downregulation of *cyclin D1* by miRNA let-7 in control of cancer cell growth [31].

The regulation of *cyclin D1* by miRNA is likely of broad importance as *cyclin D1* encodes the regulatory subunit of a kinase that phosphorylates and inactivates the pRb family proteins to inhibit DNA synthesis, and phosphorylates nuclear respiratory factor 1 (NRF-1) to inhibit mitochondria biogenesis [32, 33]. Furthermore, *cyclin D1* promotes breast epithelial cell angiogenesis and migration [34], and promotes chromosomal instability which in turn contributes to tumorigenesis [35].

The miR-221/222 cluster regulates the cell cycle, cell growth and epithelial-to-mesenchymal transition (EMT) in breast cancer. The expression of miR-221/222 is increased in HER2/neu-positive primary human breast cancer [36]. The CDK inhibitors p27^KIP1^ and p57^KIP2^ are target genes of miR-221/222. miR-221/222 inhibited p27^KIP1^ and p57^KIP2^ abundance, facilitating G_1_-S phase transition, thereby promoting cancer cell proliferation [36, 37]. Moreover, miR-221/222 may contribute to the aggressive clinical behavior of basal-like breast cancers. The breast cancer basal-like subtype-specific miRNAs, miR-221 and miR-222, promote EMT in breast cancer by targeting *TRPS1* (trichorhinophalangeal syndrome type 1) which inhibits EMT by repressing ZEB2 expression [38]. miR-221 and/or miR-222 expression in MCF-7 and T47D breast cancer cells decreased ERα expression associated with tamoxifen resistance [39].

The onco-miRNA miR-21 is overexpressed in a wide variety of cancers including breast cancer [40, 41]. miR-21 induced cellular proliferation, migration, invasion, EMT, cancer stem cell characteristics and chemotherapy resistance in human breast cancer [42, 43]. High miR-21 level is associated with poor prognosis, advanced stage, positive lymph node status and reduced survival time in breast cancer. miR-21 promotes MCF-7 cellular proliferation in part through inhibiting the expression of a tumor suppressor gene programmed cell death 4 (PDCD4) [41]. In colon cancer, miR-21 participates in a DNA damage-induced G_2_-M checkpoint through suppressing the cell cycle regulator Cdc25A [44]. A recent report demonstrated the miR-21 regulates the cell cycle through targeting Cdc25A in MCF-7 breast cancer cells [45]. With a potential anti-cancer chemical 3,3′-Diindolylmethane treatment, miR-21 expression increased, Cdc25A level decreased, and cellular proliferation was inhibited [45].

miR-27a expression is upregulated in human breast cancer cell lines [46]. In MDA-MB-231 cells, miR-27a negatively regulated the zinc finger *ZBTB10* gene and *Myt-1,* thereby promoting breast cancer cell proliferation [47]. *Myt-1* phosphorylates and inactivates cdc2/cyclin B, inhibiting the mitotic initiation of cell cycle [48]. miR-27a suppressed *Myt-1,* increased cdc2/cyclin B activity and promoted the G_2_-M checkpoint in MDA-MB-231 cells. Thus, distinct miRNAs affect key genetic targets that govern distinct cell-cycle checkpoints including cyclins, CDKs, CDK inhibitors and the G_2_-M regulation apparatus. In addition to cell-cycle control, breast tumor onset and progression and breast tumor stem cells are also regulated by distinct miRNAs.

## miRNA Regulation of Breast Cancer Stem Cells

Cancer stem cells (CSCs) are characterized by their self-renewal capacity, an ability to differentiate into non-tumorigenic cell progeny, and their ability to seed tumors when transplanted into animal hosts [49]. Cell surface markers such as CD44, CD24, CD133, epithelial-specific antigen and aldehyde dehydrogenase-1 are frequently used to isolate and enrich CSCs. CD44^+^CD24^-/low^Lineage^-^ cells were characterized as mammary gland tumorigenic CSCs [50].

The involvement of miRNAs in regulating tumor formation by CSCs or tumor-initiating cells (T-IC) has been widely investigated. Let-7 expression is very low to undetectable level in embryonic stem cells (ES cells) and increases with differentiation. The same pattern of let-7 expression was observed in breast cancer stem cells [30]. A comparison of miRNA expression between breast T-IC and non-T-IC demonstrated reduced let-7 expression in T-IC and increased abundance with differentiation [30]. Transduction of breast CSCs with let-7 reduced the proportion of undifferentiated cells, inhibited cell proliferation, mammosphere formation, and tumor formation in vivo [30].

Clarke and colleagues identified 37 miRNAs which were differentially expressed between human breast CSCs (CD44^+^CD24^-/low^lineage^-^) and lineage^-^ nontumorigenic breast cancer cells [51]. miR-200 suppresses EMT in breast cancer [52]. The miR-200 family members were downregulated in human breast CSCs and normal mammary stem/progenitor cells. Expression of miR-200 inhibited breast cancer stem cell expansion in vitro, and suppressed the tumor formation ability of human breast cancer stem cell in vivo [51].

Down-regulation of miR-34c was reported in human breast T-IC [53]. Ectopic miR-34c expression reduced breast T-ICs self-renewal, inhibited EMT and suppressed tumor cell invasiveness via silencing Notch4 [53]. Zhu et al. found the reduced miR-128 expression in human breast T-IC was accompanied by Bmi-1 and ABCC5 overexpression, and associated with chemotherapeutic resistance and poor survival [54]. Enforced miR-128 expression increased the sensitivity of breast cancer cells to doxorubicin-induced apoptosis and DNA damage [54].

Emerging evidence has demonstrated the importance of CSCs in cancer initiation, cancer metastasis and drug resistance. CSCs are believed to be one of the most promising targets for cure of cancer. The discovery that non-coding RNAs regulate CSCs widens our understanding of CSCs, and may provide potential novel strategies for breast cancer therapy.

## Other Non-coding Small RNAs in Breast Cancer

SnoRNAs primarily guide chemical modifications of other RNAs. Deletion of chromosome 6q, including region 6q14-q16, is frequently observed in breast cancer. The small non-coding snoRNA U50 is a candidate tumor suppressor gene in the 6q14-16 region, playing a role in the development and/or progression of breast cancer [55]. Genomic deletion and transcriptional downregulation of snoRNA U50 was detected in breast cancer cell lines. Re-expression of snoRNA U50 inhibited colony formation of the human breast cancer cells Hs578t and MDA-MB-231.

piRNAs are small non-coding RNA that form RNA-protein complexes through interactions with piwi proteins. piRNA was initially discovered in germ line cells, and considered as germ line-specific small RNAs [56]. Emerging evidence indicates that piRNA expression occurs in somatic cells [57, 58] and piwil2 expression has been identified in human breast cancer cells [59]. High-throughput deep sequencing identified a group of small RNAs matching piRNA sequences in human breast cancer tissues and breast cancer cell lines [60].

The study of these non-coding small RNAs in human cancer is just starting. The mechanisms regulating snoRNAs and piRNAs in human breast cancer remain unknown. The identification of the expression signature of these non-coding small RNAs in breast cancer subtypes, and an understanding of their functional significance to oncogene expression, tumor initiation and tumor cell metastasis may shed important new perspectives on the role of these non-coding small RNAs in breast cancer.

## Therapeutic Application of miRNA

Small non-coding RNA-based diagnostic and therapeutic applications for human cancer are expected in the near future. Although tumor-targeted delivery and local administration are still major challenges to the practical application of gene therapy for cancer, miRNA-based cancer therapeutic approaches are being established and tested in animal models. Synthetic miRNA mimics or miRNA expression vectors have been successfully applied to restore or overexpress miRNA in vitro. Chemically modified LNA anti-sense miRNA inhibitor and other approaches have been used to block miRNA function in cells. Kim et al. recently reported significant anti-tumor effect of virus-mediated delivery of miR-145 combined with 5-FU to treat breast cancer [61]. Intranasal delivery of let-7 [62] and intravenous delivery of miR-34a mimics [63] for non-small-cell lung cancer treatment and a virus-mediated delivery of *miR-26a* for liver cancer treatment in mouse model [64] demonstrate the promise of miRNA for treatment of cancer.

## Concluding Remarks

Dysregulated expression of miRNAs has implicated components of the non-coding genome as either oncogenes or tumor suppressors of breast cancer. Experimental evidence has shown specific miRNAs regulating the initiation, progression, metastasis and drug resistance of breast cancer via control of the cell cycle, altering cellular proliferation, altering cellular apoptosis and/or controlling the population of tumor stem cells. Dysregulated miRNA expression has also been observed in cancer associated fibroblasts (CAF) and in the systemic circulation [65, 66]. The circulating miRNAs have the potential to serve as novel diagnostic and prognostic biomarkers for breast cancer. A specific subset of dysregulated miRNAs in breast cancer cells may serve as targets for gene therapy either alone or as an adjuvant treatment to current clinical protocols for breast cancer patients.

## Abbreviations

siRNA: small interfering RNA
miRNA: microRNA
piRNA: piwi-interacting RNA
snoRNA: small nucleolar RNA
EMT: epithelial-to-mesenchymal transition
T-IC: tumor-initiating cells
CSC: cancer stem cells
CDK: cyclin-dependent kinase
CAF: cancer associated fibroblast
